# Expression Levels of an Alpha-Synuclein Transcript in Blood May Distinguish between Early Dementia with Lewy Bodies and Parkinson’s Disease

**DOI:** 10.3390/ijms22020725

**Published:** 2021-01-13

**Authors:** Laura Marsal-García, Aintzane Urbizu, Laura Arnaldo, Jaume Campdelacreu, Dolores Vilas, Lourdes Ispierto, Jordi Gascón-Bayarri, Ramón Reñé, Ramiro Álvarez, Katrin Beyer

**Affiliations:** 1 Department of Pathology, Germans Trias i Pujol Research Institute (IGTP), Universitat Autònoma de Barcelona (UAB), 08193 Barcelona, Spain; laauraa.mg@gmail.com (L.M.-G.); aurbizu@igtp.cat (A.U.); larnaldo@igtp.cat (L.A.); 2 Servei de Neurologia, Hospital Universitari Bellvitge, 08907 L’Hospitalet de Llobregat, Spain; jcampdelacreu@bellvitgehospital.cat (J.C.); jordigneuro@bellvitgehospital.cat (J.G.-B.); rrene@bellvitgehospital.cat (R.R.); 3 Servei de Neurologia, Hospital Universitari Germans Trias i Pujol, 08916 Badalona, Spain; dvilas.germanstrias@gencat.cat (D.V.); mlispierto.germanstrias@gencat.cat (L.I.); ralvarez.germanstrias@gencat.cat (R.Á.)

**Keywords:** alpha-synuclein, SNCA transcript variants, dementia with Lewy bodies, Parkinson’s disease, differential mRNA transcript expression, peripheral biomarker, disease progression

## Abstract

Lewy body diseases (LBD) including dementia with Lewy bodies (DLB) and Parkinson disease (PD) are characterized by alpha-synuclein pathology. DLB is difficult to diagnose and peripheral biomarkers are urgently needed. Therefore, we analyzed the expression of five alpha-synuclein gene (*SNCA*) transcripts, SNCAtv1, SNCAtv2, SNCAtv3, SNCA126, and SNCA112, in 45 LBD and control temporal cortex samples and in the blood of 72 DLB, 59 PD, and 54 control subjects. The results revealed overexpression of SNCAtv1 and SNCA112 in DLB, and SNCAtv2 in PD temporal cortices. In DLB blood, diminution of all *SNCA* transcripts was observed. SNCAtv1 and SNCAtv2 were diminished in PD with disease onset before 70 years. SNCAtv3, driven by its own promoter, showed opposite expression in early DLB and PD, suggesting that its amount may be an early, DLB specific biomarker. Correlation between blood transcript levels and disease duration was positive in DLB and negative in PD, possibly reflecting differences in brain alpha-synuclein aggregation rates associated with differences in disease courses. In conclusion, SNCA transcripts showed a disease-specific increase in the brain and were diminished in blood of LBD patients. SNCAtv3 expression was decreased in early DLB and increased in early PD and could be a biomarker for early DLB diagnosis.

## 1. Introduction

Dementia with Lewy bodies (DLB) and Parkinson’s disease (PD) are Lewy body disorders (LBD). They are characterized by Lewy body pathology (LBP) in the brain [1], and neuropathologically, two forms of LBP can be distinguished. In common LBP, a mixture of LBP and concomitant Alzheimer disease (AD) pathology can be found, and in pure LBP, the brain is mainly affected by this type of pathology [2]. LBP consists of abnormal intraneuronal aggregates of alpha-synuclein (AS), so called Lewy bodies (LBs), and Lewy neurites [1]. In PD, these appear first in the substantia nigra [3,4], and in DLB an early widespread distribution throughout the brain is found [5]. Cases with pure or common LBD cannot be distinguished clinically. In DLB, the first clinical symptoms are linked to dementia with motor signs appearing concomitantly or less than one year after onset of cognitive symptoms [6]. Additionally, the disease often progresses rapidly with a duration of between six to 10 years [6,7]. On the contrary, the cognitive decline in PD develops usually late and patients can survive up to 30 years [8,9]. Although DLB and PD dementia (PDD) share similar biological substrates and may represent subtypes of the LBD continuum, some neuropathological differences have been identified [10]. These include a more severe and extended AS load in the cortex and striatum, and higher tau load in the medial temporal cortex in DLB than in PDD. The AS load in the hippocampus is also different, and whereas CA 1/2 is severely involved in DLB, CA 2/3 is more frequently affected in PDD. The pattern of neuronal loss in the substantia nigra also differs between DLB and PDD, and whereas no pedunculopontine cholinergic loss is observed in DLB, it occurs in PDD with hallucinations [10]. DLB is also characterized by the clinical overlap with AD due to the concomitant AD pathology in DLB brains leading to the misdiagnosis of up to 80% of DLB cases [11]. Misdiagnosis results in the administration of incorrect treatments that can adversely affect cognition of DLB patients [6]. Therefore, the identification of biomarkers for the differential diagnosis of DLB is of paramount importance. Current fluid biomarkers for AD are reduced Aβ42 (<600 pg/mL) and elevated total and phosphorylated tau (>275 pg/mL and >50 pg/mL, respectively) in cerebrospinal fluid (CSF) [12,13]. Their suitability for differential DLB diagnosis has been also assessed but no conclusive results have been obtained [14], and no peripheral DLB biomarkers have been identified so far. 

As the main component of LBs, AS is now recognized as a key element driving the development of LBD [15]. It is a small, unfolded, and multifunctional protein [16] and its increase in vulnerable brain areas has been linked to disease development [17,18]. AS gene (SNCA) duplication and triplication have been reported as causative factors for the development of PD [19,20] and this dose duplication is directly responsible for elevated SNCA expression levels in the brain [21]. 

The study of AS expression in CSF in PD revealed increased levels of AS, also in early disease stages [22,23]. Additionally, reduced AS levels have been found in the serum of DLB patients compared to both AD and controls, and the increase of AS in plasma associated with the progression of PD has been reported [24]. 

The *SNCA* gene undergoes complex splicing events (recently reviewed in [25]). On one hand it causes variation in the exon content of its 5′-untranslated region (UTR), bearing transcripts driven by at least four different promoters. On the other hand, additional in-frame splicing of two exons gives rise to a large number of *SNCA* transcripts [26]. Moreover, the length of the 3′UTR comprising the inclusion or not of certain miRNA binding sites has been also described to vary in the various *SNCA* transcripts [27].

Only a few studies have addressed the differential expression of the different *SNCA* transcripts in both brain and blood. Although the suitability of total SNCA mRNA expression changes as diagnostic or progression biomarker has been studied for PD, little information is available for DLB. In the present study, we determined SNCA transcript variant expression in brain and blood of DLB and PD patients compared to controls with the aim to find out if (i) the SNCA expression pattern differs between DLB and PD brain and blood, (ii) there exists a correlation between SNCA transcript expression and clinical parameters, and (iii) DLB and PD share correlation tendencies or not. Therefore, we analyzed the differential expression of five SNCA transcript variants (tv), SNCAtv1, tv2, tv3, SNCA126 and SNCA112 in the temporal cortex and blood of DLB and PD patients, and control individuals.

## 2. Results

### 2.1. Characteristics of Post-Mortem Cases

All 32 cases with neuropathological changes presented neocortical LBs and had been diagnosed with DLB or PD corresponding to their clinical characteristics. Clinical characteristics including disease onset, disease duration, and male/female ratio are shown in Table 1. Age at death was significantly lower in control than in patients. However, when taking into account the disease duration in the other groups, and when comparing the age at disease onset of LBD and age at death of controls, no significant difference was found (*p* = 0.235). The male/female ratio did not show significant differences between the three groups. Disease onset was at a significantly younger age and disease duration was significantly longer in PD than in DLB cases. These differences represent intrinsic characteristics of both diseases. 

### 2.2. Demographic and Clinical Data of Participants

Demographic and clinical data of patients are shown in Table 2. Mean age was similar in DLB patients and control subjects (CTRLs), according to the inclusion criteria; however, PD patients were significantly younger (*p* = 0.021). The male-female ratio was higher in PD and DLB compared to CTRLs, but no gender-specific expression changes were observed during data analyses. Similar to post-mortem cases, the age at disease onset was significantly lower in PD than in DLB. Since these cohorts were being recruited during routine clinical practice and none of them was an end-stage patient, disease duration was similar in the patient groups (Table 2). Family history of dementia was reported in 11.1% of DLB patients and of PD, in 27.2% of PD patients.

### 2.3. Relative SNCA Isoform Expression Levels in Different Brain Regions and Blood 

To ascertain which of the SNCA transcripts was mostly expressed in frontal and temporal cortices, caudate nucleus, pons and blood, Ct values obtained by real-time PCR were compared. After corrections for the different efficiencies and thresholds for each PCR reaction, the estimation of differences between expression levels was determined considering the overall SNCA expression as 100% (Table 3). The results revealed that SNCAtv1 was the main transcript in all brain region and blood, representing about 60% of the transcripts analyzed here. SNCAtv2, a transcript that at least in part shares the SNCAtv1 promoter, showed two to three times less expression than SNCAtv1. The initial exon of SNCAtv3 is located upstream to the initial exons of SNCAtv1 and SNCAtv2. Correspondingly, it is driven by different regulatory elements than the two main transcripts and represents about 15% of SNCA transcripts in the cortex and blood, and less than 10% in other brain areas. SNCA112 and SNCA126 are transcripts generated by in-frame splicing and represent only approximately 1% of the SNCA mRNAs analyzed here (Table 3). 

### 2.4. SNCA Transcript Expression in the Temporal Cortex

**Figure 1:**
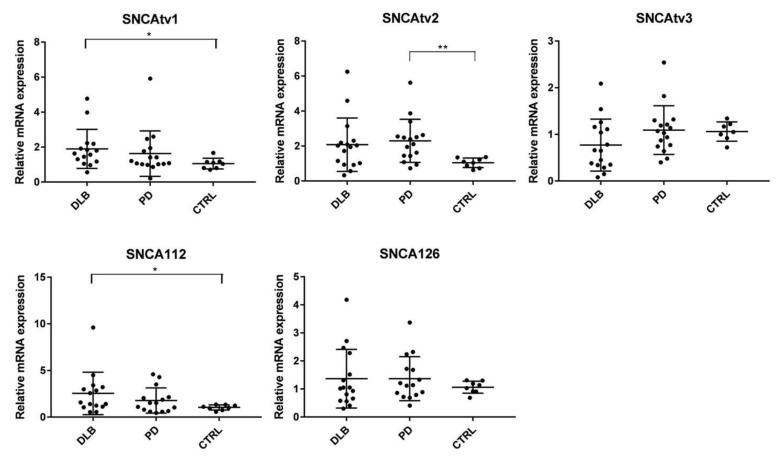
Expression of SNCA transcript variants in the temporal cortex of DLB and PD patients and controls. * *p* < 0.05, ** *p* < 0.01.

The expression levels of the five SNCA transcripts were determined in the temporal cortex of DLB and PD patients, and in controls. The results revealed disease-specific expression changes, which are shown in Figure 1. SNCAtv1 was significantly overexpressed in DLB compared to controls, and a slight, but not significant increase was observed in PD (Figure 1). SNCAtv2 was increased in both DLB and PD, However, it was only significant in PD; in DLB, because of the high standard deviation range, it did not reach significance. No significant expression changes were detected for either SNCAtv3 or SNCA126. The levels of SNCA112 were significantly increased in DLB, although a high standard deviation range was also detected. In PD temporal cortices, SNCA112 levels did not show a significant expression change, though a tendency to increase was observed (Figure 1). 

### 2.5. Correlation between the Expression of SNCA Transcript Variants in Brain

To find out if there was a correlation between the different SNCA transcripts in the temporal cortex of LBD, all LBD-cases were grouped, and the Pearson R-Score was determined. The results are shown in Table 4 and reveal a strong positive correlation (R > 0.7) between the major transcript SNCAtv1 and SNCAtv2, between SNCAtv1 and the minor transcripts SNCA126 and SNCA112, between SNCAtv2 and the minor transcripts, and between SNCA126 and SNCA112. On the contrary, the correlation between SNCAtv3 and all other transcripts was negative (Table 4). 

### 2.6. SNCA Transcript Expression in Blood

**Figure 2:**
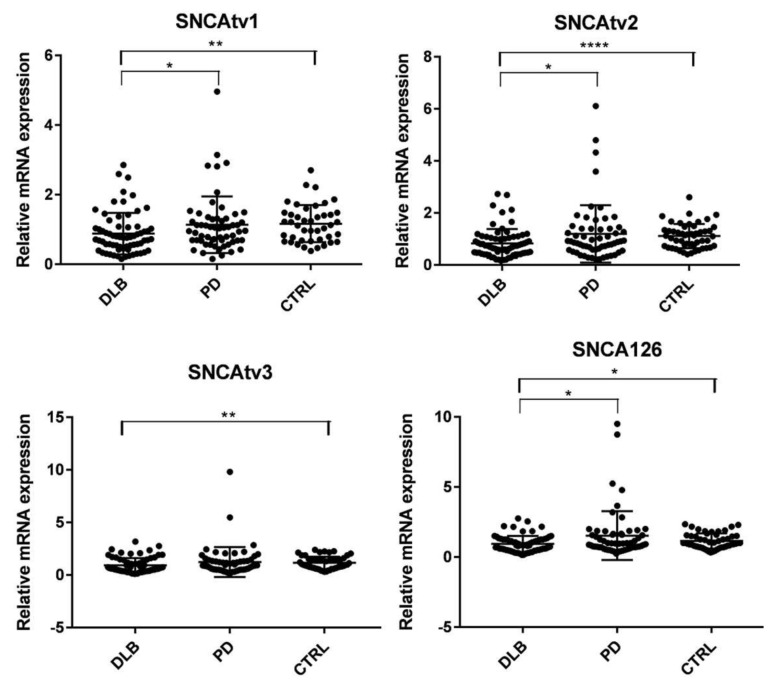
Expression of SNCA transcript variants in blood of DLB and PD patients, and controls. * *p* < 0.05, ** *p* <0.01, **** *p* <0.001.

The expression of only four of the five SNCA transcripts could be determined in blood. SNCA112 had the lowest expression, and the construction of a valid standard curve failed. Figure 2 shows that compared to controls, the expression of all four SNCA transcripts was significantly diminished in DLB, and remained unchanged in PD. Down-regulation of SNCAtv1, SNCAtv2, and SNCA126 was also significant when compared with PD (Figure 2).

### 2.7. Correlation between SNCA Transcript Expression, Disease Duration and Age-at-Onset in Blood

Significant, moderate (R > 0.3) and strong (R > 0.7), positive correlation was observed between all transcripts in blood of both DLB and PD patients (Table 5). 

**Figure 3:**
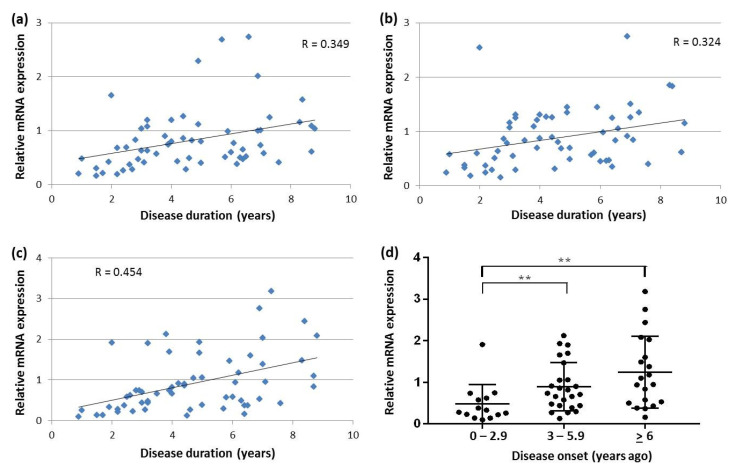
SNCA transcript expression in blood of DLB patients: (**a**) correlation between SNCAtv2 and disease duration; (**b**) correlation between SNCA126 and disease duration; (**c**) correlation between SNCAtv3 and disease duration; (**d**) comparison of SNCAtv3 expression levels in three DLB groups with different disease duration. ** *p* <0.01.

The analysis of correlation between disease duration and SNCA transcript expression in DLB revealed that the shorter the duration from DLB onset, the lower the SNCA transcript expression levels in the blood (Figure 3a–c). Whereas this positive correlation was not significant for SNCAtv1 (R = 0.231, *p* = 0.09), it was the strongest for SNCAtv3 with a correlation coefficient of almost 0.5 (Figure 3c). To further investigate the effect of disease duration on SNCAtv3 expression, DLB patients were divided into three groups: (i) patients with disease duration between 0 and 2.9 years (*n* = 14), (ii) patients with disease duration between 3 and 5.9 years (*n* = 25), and (iii) patients with disease duration of 6 years or more (*n* = 20). The results showed that SNCAtv3 levels were significantly lower in patients who recently debuted with DLB when compared to patients who were suffering from the disease between 3 and 5.9 years, and even lower when compared with patients with a DLB course of 6 years or more (Figure 3d). 

**Figure 4:**
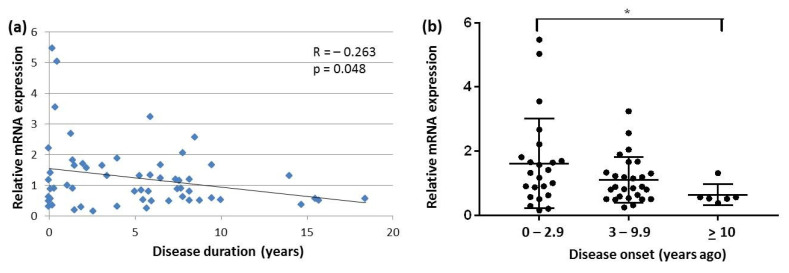
SNCAtv3 expression in blood of PD patients: (**a**) correlation between SNCAtv3 expression and disease duration; (**b**) comparison of SNCAtv3 expression levels in three PD groups with different disease duration. * *p* < 0.05.

In contrast to DLB, in PD, a negative correlation was found between SNCA transcript expression and disease duration. This correlation was significant in the case of SNCAtv3 (Figure 4a). When dividing PD patients into three groups: (i) patients with disease duration between 0 and 2.9 years (*n* = 23), (ii) patients with disease duration between 3 and 9.9 years (*n* = 27), and (iii) patients with disease duration of 10 years or more (*n* = 6), SNCAtv3 levels were significantly lower in patients with long disease duration compared to patients with recent disease onset (Figure 4b). Finally, when comparing SNCAtv3 expression levels of the recent DLB onset group (0.48 + 0.47) with those of the corresponding PD group (1.62 + 1.37), SNCAtv3 was significantly diminished in DLB (*p* < 0.001)

**Figure 5:**
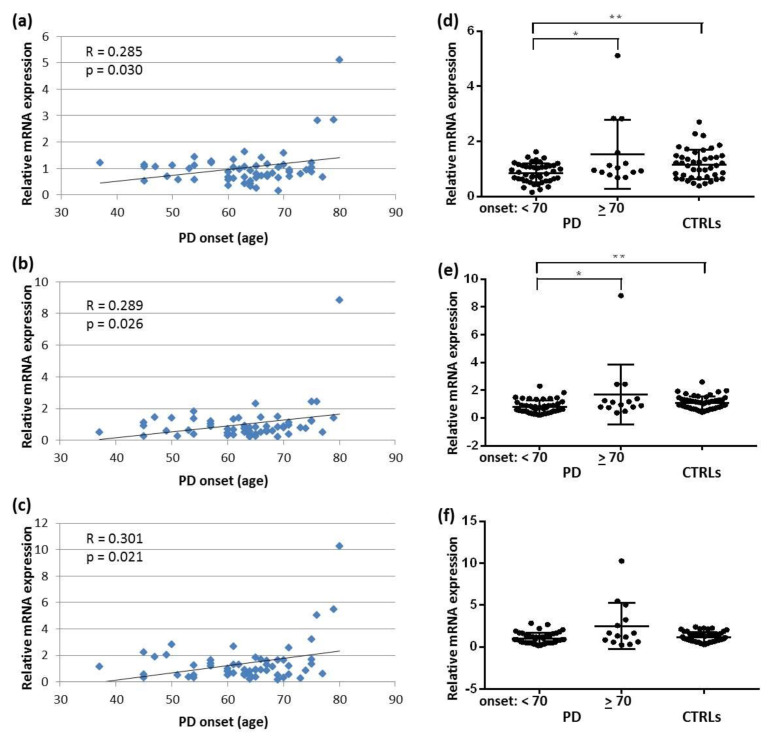
Correlation between the age of PD onset and (**a**) SNCAtv1, (**b**) SNCAtv2, and (**c**) SNCAtv3 expression. SNCA expression in two age-at-onset dependent subgroups compared to controls: (**d**) SNCAtv1; (**e**) SNCAtv2; (**f**) SNCAtv3. * *p* <0.05, ** *p* < 0.01.

Although no correlation between SNCA transcript expression and disease onset was found in DLB, in PD significant weak (R < 0.3) and moderate positive correlations between age at disease onset and SNCAtv1, SNCAtv2, and SNCAtv3 were identified (Figure 5a–c). Therefore, we divided the group of PD patients by their age at disease onset: 44 patients presented with the first disease symptoms before the age of 70 years, and 14 at 70 years or later. The comparison of SNCA transcript expression levels between both groups and controls revealed that the expression of SNCAtv1 and SNCAtv2, but not of SNCAtv3, was significantly higher in PD patients who presented first symptoms before the age of 70 years in comparison with both PD patients who debuted at 70 years or later, and controls (Figure 5d–f). 

## 3. Discussion

In the present study we analyzed the expression of five *SNCA* transcripts in brain and blood of patients diagnosed with DLB or PD. SNCAtv1 was identified as the major *SNCA* transcript representing between 50 and 60% of total *SNCA*. The initial exon of SNCAtv2 follows the initial exon of SNCAtv1 without intron in between indicating that both transcripts are partly co-regulated [25]. The initial exon of SNCAtv3 is located about 1 kb up-stream to the other initial exons and therefore, its independent regulation can be expected. Two minor transcripts, SNCA112 lacking exon 5, and SNCA126 lacking exon 3 are generated as the result of in-frame splicing.

In the temporal cortex, disease-specific *SNCA* transcript expression changes were found. Whereas SNCAtv1 was overexpressed in DLB, SNCAtv2 was increased in PD. SNCAtv1 is the major *SNCA* transcript representing about 50% of overall SNCA mRNA in the temporal cortex, suggesting that the increase of AS and its modified species may be due to increased expression of SNCAtv1 [31]. In contrast, SNCAtv2 only represents about 20% of total *SNCA* mRNA in the temporal cortex. Correspondingly, a much lower AS increase should be the result. In fact, in PD, the temporal cortex is affected after several years of disease progression [9,10] and AS overexpression has been reported especially in early affected areas, such as the sustantia nigra pars compacta [32].

Furthermore, we found a strong positive correlation between SNCAtv1 and SNCAtv2, as well as between both and the minor transcripts SNCA126 and SNCA112. Instead, SNCAtv3 expression levels correlated negatively with all other transcripts. These results confirm, that SNCAtv3 expression is driven by a different promoter than SNCAtv1 and SNCAtv2, and that the latter share similar regulatory elements. Additionally, the results indicate that the minor transcripts SNCA126 and SNCA112 are mainly generated from SNCAtv1 and SNCAtv2, but not from SNCAtv3.

Moreover, SNCA112 was overexpressed in DLB confirming the results we previously published for frontal cortices from DLB patients. In that study, we showed an important increase of SNCA112 in DLB cases which presented pure and not common LB pathology in the brain [33]. As the result of exon 5 splicing out, SNCA112 gives rise to AS112, a 112-amino acid protein lacking C-terminal residues 103–130 [34]. This deletion occurs in the less organized part of the protein bearing a protein variant with increased aggregation propensity [35]. Correspondingly, it has been shown that the aggregation rate of alpha-synuclein increases after the introduction of nucleation seeds composed of AS fibrils into AS expressing cells [36]. This seeding process is tightly linked to the formation of soluble intermediates that precedes the formation of toxic AS species, and their propagation throughout the brain [37,38]. Although SNCA112 represents less than 0.5% of total *SNCA* in the temporal cortex, only doubling its concentration might increase the formation of seeding fibrils, and together with total AS increase initiate and accelerate the aggregation process. So far, AS112 could not be detected in brains affected with LB pathology, but the fact that it only represents a very small fraction of total AS and that it could be sequestered instantaneously to form seeding fibrils, converts its detection into a challenge. 

In blood, all four *SNCA* transcripts were significantly diminished in DLB compared to controls. In a recent study, Funahashi and colleagues analyzed *SNCA* expression levels in the blood of 20 DLB patients and 20 controls [39]. SNCA expression was analyzed for all transcripts containing all coding exons together and no expression changes were found. However, in contrast to our results, SNCA126 expression was increased. This difference could be due, on one hand, to the small sample number included in Funahashi’s study, or on the other, to ethnical differences. Additional studies in both, the Asian and Caucasian populations should confirm this difference.

In our PD cohort, the expression of *SNCA* transcript variants did not differ significantly when compared to controls. Nevertheless, a positive correlation between *SNCA* transcript expression and disease onset was found. Correspondingly, the expression of SNCAtv1, SNCAtv2, and SNCA126 was diminished in PD patients who debuted with PD symptoms before the age of 70 years in comparison with those with later disease onset. This division of PD patients in dependency on their age at onset further revealed that SNCAtv1 and SNCAtv2 were significantly diminished in the earlier onset group when compared to controls. In a recent study, Locascio and colleagues analyzed *SNCA* transcript levels in three independent PD cohorts and found reduced *SNCA* mRNA at early PD stages [40]. Here, we found the diminution of SNCAtv1 and SNCAtv2, which together represent about 80% of total *SNCA* in blood. This diminution could correspond to Locascio’s findings, who analyzed the expression of all coding exon-containing *SNCA* variants together. Since in the present study SNCAtv3 was analyzed independently, the decrease of SNCAtv1 and SNCAtv2 could be detected in all PD patients with onset before 70 years, independently on their disease stage. 

Two additional studies analyzed *SNCA* in blood of PD patients, both in correlation with *SNCA* promoter methylation [41,42], and in both hypo-methylation of the *SNCA* promoter, but no significant expression changes of SNCA mRNA was found. It is important to take into account that mainly SNCAtv1 and SNCAtv2 are regulated by methylation changes in the CpG-island in *SNCA* intron 1 [43]. Together, the results of our studies and the previous studies mentioned here underline the importance of analyzing SNCA transcripts with different initial exons independently, especially when seeking for a disease biomarker. The fact that these transcripts are driven by different promoters and that SNCAtv3 expression differs from SNCAtv1 and SNCAtv2 suggest that SNCAtv3 may mask expression changes of the individual transcripts. 

Furthermore, another recent study analyzed AS levels in blood cells from PD patients with and without dementia in comparison with controls [44]. The results revealed that total AS was increased in PD patients without dementia in comparison with PD patients with dementia. Since dementia in PD usually develops at late disease stages, these results are in concordance with our observation that the lowest *SNCA* levels are found at late disease stages and that *SNCA* is increased at the earlier stages. 

Although SNCAtv1 and SNCAtv2 expression was diminished in both DLB and PD with onset before 70 years, SNCAtv3 expression did not differ between the entire DLB and PD cohorts compared to controls. However, its expression correlated inversely with disease duration in both diseases, and opposite SNCAtv3 expression levels, as a decrease in early DLB and an increase in early PD were observed. Since both DLB and PD are synucleinopathies sharing similar neuropathological features in the brain, this result has been rather unexpected. A possible explanation could be the differences in the clinical course between both diseases, because whereas DLB is a rapidly progressing disease of short duration [6,7], PD usually has a much slower but longer disease course [9]. This difference could have a direct effect on AS oligomerization and aggregation rates in the brain, which at the same time could be differently reflected in blood. Furthermore, different spreading pathways of brain alpha-synuclein pathology have been recently proposed [45]. According to this hypothesis, in PD brain, AS spreading would start in the brainstem and reach the cortex relatively late. In contrast in DLB, AS spreading would start in the olfactory bulb and extend directly to the cortex. These differences could also be reflected as SNCAtv3 expression changes in blood, which could represent a biomarker to differentiate between early DLB and PD. In addition, the suitability of SNCAtv3 as an early DLB biomarker should be further explored, since it could be useful to identify those patients in prodromal cohorts who will develop DLB in the future. 

The main weakness of our study is the relative low number of participants, which made it impossible to further subdivide the cohorts, especially the PD cohort. In the case of PD, the analysis of the results in different PD subtypes would permit to obtain more precise results on the suitability of *SNCA* transcripts as disease biomarker. However, the study has been performed over several years and therefore robust expression data have been obtained. Nevertheless, the results must be validated in additional and independent cohorts and in different laboratories. 

Further research is needed to answer several questions that have arisen. First, are changes in *SNCA* transcript expression contrasting in different PD subtypes? Second, does hypo-methylation of the CpG island in *SNCA* intron 1 correlate with expression changes of individual transcripts, specifically SNCAtv1 and SNCAtv2? Third, is SNCAtv3 a valid biomarker to distinguish between individuals with idiopathic REM sleep behavior disorder who will convert to DLB from those who will convert to PD? If so, prodromal patients could be specifically identified and be eligible for the study of preventive treatments. 

In conclusion, our study highlights the importance of analyzing the different SNCA transcript variants instead of total SNCA in biomarker research. To determine the biomarker potential of the three major SNCA transcripts, SNCAtv1, tv2 and tv3 to predict disease progression, these should be analyzed in longitudinal studies including both PD and DLB cohorts with a detailed clinical record. Furthermore, the determination of SNCA transcript expression levels in prodromal LBD patient cohorts may provide information on disease development in the future. 

## 4. Materials and Methods 

### 4.1. Source of Tissues

Post-mortem brain samples with their corresponding clinical and neuropathological diagnoses were provided by the Institute of Neuropathology Brain Bank and the Neurological Tissue Bank of the University of Barcelona/Hospital Clinic, Barcelona, Spain. Brain samples were obtained from 16 donors with DLB, from 16 donors with PD, and 13 donors devoid of neurological signs or symptoms and lack of neuropathological findings. Temporal cortex samples, obtained from all DLB, and PD, and 11 control brains were used for the study of differential *SNCA* transcript expression. Samples from the control frontal cortex, caudate nucleus and pons were used to analyze the relative amount of SNCA transcripts in the different brain regions. The presence and distribution of AS related pathology was evaluated following the criteria established by the third report of the DLB consortium [30] and according to Braak and Braak [29]. AD-type pathology was assessed by using the Braak and Braak criteria [28]. The study was carried out in accordance with the requirements of the local Ethics Committee. 

### 4.2. Participants

A total of 185 individuals were included from two different hospitals: Hospital Universitari Germans Trias i Pujol (Badalona, Barcelona) and Hospital Universitari de Bellvitge (L’Hospitalet de Llobregat, Barcelona). Three cohorts were recruited: 

DLB patients. A total of 72 patients who fulfilled criteria for probable DLB [6,30] were prospectively recruited from those visited in the Neurodegenerative disease Unit of both centers as routine clinical practice.

PD patients. A group of 59 PD patients diagnosed according to the UK PD Society Brain Bank criteria [46] were included. None of these patients presented cognitive impairment, which was defined as subjective cognitive complaints, based on the patient’s and informant interview, and on the Minimental State Examination (MMSE) score, considering cognitive impairment if the MMSE punctuation was <24 points. MMSE testing forms, part of the routine protocol used during the initial cognitive status screening, although the use of other tests, such as the Montreal Cognitive assessment (MoCA) may detect cognitive decline with higher sensitivity.

In DLB and PD patients, age at onset was defined as the age when memory loss or parkinsonism was first noticed by the patient or his/her relatives. 

Control subjects. A total of 54 CTRLs were selected among non-blood relatives of the patients, age-matched with the DLB group.

The study was carried out with the approval of the local Ethical Committees for Clinical Investigation of the institutions involved in the study, and a written informed consent was signed by all participants or their legal guardians according to the Declaration of Helsinki. 

### 4.3. RNA Purification, Reverse Transcription, and Assessment of mRNA Stability

From brain tissue, RNA was isolated with TRI-Reagent (MRC, Cincinnati, OH, USA) according to the manufacturer’s protocol. Since red blood cells are the major source of AS in blood [46], whole blood samples were used for expression analyses. RNA isolation was carried out after collection of 3 mL of whole blood in PAXgene Blood RNA tubes (PreAnalytiX, Hombrechtikon, Switzerland) by the use of the PAXgene Blood RNA Kit 50, v2 (PreAnalytiX). RNA quantity, purity and integrity were ascertained by the Agilent 2100 Bioanalyzer (Agilent Technologies, Santa Clara, CA, USA). For RNA obtained from blood, all RIN values were higher than 8. In regard to brain samples, only RNAs with RIN higher than 6 were stored at −80 °C until use [47].

For brain samples, 2 μg and for blood samples, 1 μg of total RNA was used for reverse transcription by Ready-to-go™ You-Prime First-Strand Beads (GE Healthcare, Buckinghamshire, UK). mRNA degradation rates of all *SNCA* transcript variants were assessed in order to assure that they presented similar stabilities even at RIN values of about 6, as described before [48]. 

### 4.4. Primer Design 

**Figure 6:**
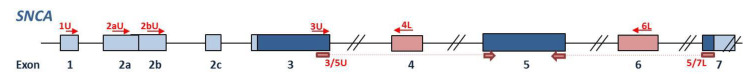
Schematic representation of the *SNCA* gene showing the localization of the primers listed in Table 6. Arrows represent the location of the primers.

Primer sequences and primer combinations are given in Table 6 and their location is shown in Figure 6. The *SNCA* gene bears numerous transcript variants by the inclusion of alternative 5’ initial exons and by splicing out of internal exons. The five transcripts analyzed here present specific sequences permitting the design of isoform-specific primers. To amplify SNCAtv1, SNCAtv2, and SNCAtv3, the forward primer was located in exons 2a, 2b, and 1, respectively. A common reverse primer was located in exon 4 (Figure 6). All exon 4 lacking transcripts giving rise to AS126 were co-amplified by the use of a forward primer that spanned the boundary between exons 3 and 5, and a reverse primer was located in exon 6. Exon 6 lacking transcripts giving rise to AS112 were also co-amplified, the forward primer as located in exon 4 and the reverse primer spanned the boundary between exons 5 and 7 (Figure 6, Table 6). 

### 4.5. Real Time PCR 

Relative expression of five *SNCA* transcript variants, SNCAtv1, SNCAtv2, SNCAtv3, SNCA126, and SNCA112 was determined in the temporal cortex and blood of all samples obtained for the study. The relative expression of these transcript variants was additionally determined in the frontal cortex, pons and caudate nucleus samples of five controls. Real-time PCR was carried out on a Rotor-Gene 6000 (Corbett Life Science, Qiagen, Hilden Germany). PCR was performed in 15 μL reactions with the QuantiTect SYBR Green PCR Kit (QiaGen), containing 16 pmol of each primer (Table 6) and 1 μL of cDNA. Since in the brain, SNCA shows mainly neuronal expression, human neuron-specific enolase 2 (*NSE*) and synaptophysin (*SYP*) were included as housekeeping genes to correct for neuronal loss in the tissue with LBD [48]. In blood, two housekeeping genes were analyzed to assess relative SNCA isoform expression, beta-actin (*ACTB*) and porphobilinogen deaminase (*PBGD*) [49].

All assays included two replicates of each sample. They were performed twice and independently to assure their reproducibility and minimize possible errors. Standard curves for the target as well as reference genes were generated for each run by amplifying the same serially diluted cDNA control sample. Relative transcript variant expression data were calculated by the ΔΔCt method. Differences between the expression amounts of the five SNCA transcripts were calculated by 2^n, n corresponding to the cycle number difference between Ct of one of the less expressing transcripts and Ct of the major transcript.

### 4.6. Statistical Analysis 

Possible differences in quantitative variables (age at disease onset, age at death and disease duration) between the pathological groups were assessed by the Kruskal-Wallis test. Fisher’s exact test was used to analyze qualitative variables. Expression analyses were performed independently for both housekeeping genes, and the normalization factor was derived from the geometric mean of this data [50]. ΔCt was obtained for all samples, and the mean of delta Ct values from the control group was used to calculate the ΔΔCt-value and the corresponding expression change for each sample. In the text, the results are shown as means and standard deviation. The significance of possible differences in the expression levels was analyzed by the Wilkoxon-Mann-Whitney test [51], and Mann-Whitney using GraphPad Prism version 8.0.0 for Windows (GraphPad Software, San Diego, CA, USA). Data distribution pattern was analyzed by the Kolmogorov-Smirnov Test of Normality. The correlation between expression levels of the five transcripts, as well as between *SNCA* transcript expression and clinical variables was determined by linear regression. Since normal distribution of the data was found, Pearson correlation coefficient R and its corresponding p-value were calculated.

## Figures and Tables

**Table 1:** Clinico-neuropathological characteristics of LBD patients and controls.

	DLB (*n* = 16)	PD (*n* = 16)	CTRLs (*n* = 11)	*p* ^1^
Mean age at death (age range)	77.2 (60–90)	79.9 (71–88)	69.9 (59–81)	0.004
Gender (male/female ratio)	1.3:1	1:1	1:1.2	0.329
Disease onset ^2^	70.0 (56–83)	59.9 (49–73)	-	<0.001
Disease duration (range)	6.1 (2–11)	18.6 (3–29)	-	<0.001
PM ^3^, hours (range)	9:00 (3:15–21:00)	8:10 (3:30–17:10)	8:30 (2:15–23:30)	0.571
AD-stage ^4^	0-IV; A-C	0-IV; A-C		
PD-stage ^5^		Braak 4–6		
AS pathol ^6^ neocortical (*n*, %)	12 (75)			
AS pathol limbic (*n*, %)	4 (25)			

All ages and disease duration are expressed in years. ^1^ *p*, obtained by the Kruskal-Wallis test; ^2^ from the beginning of cognitive symptoms for DLB, and motor symptoms in the case of PD; ^3^ post-mortem time; ^4^ AD stages following Braak and Braak, I-VI: neurofibrillary tangles, A–C: amyloid plaques [28]; ^5^ PD stages [29]; ^6^ presence and distribution alpha-synuclein related by criteria of the third DLB consortium [30].

**Table 2:** Demographic and clinical data of the participants of the study.

	DLB (*n* = 72)	PD (*n* = 59)	CTRLs (*n* = 54)	*p* ^1^
Mean age (age range)	76.0 (58–91)	67.7 (45–84)	71.6 (51–88)	0.014
Gender (male/female ratio)	1.5:1	1.3:1	1:1.5	0.033
Disease onset	71.4 (57–86)	62.3 (40–80)	-	<0.001
Disease duration ^2^ (range)	4.7 (0.9–8.7)	5.4 (0.0–18.4)	-	0.086
MMSE ^3^, mean (range)	15.1 (3–28)	-	-	-
UPDRS-III ^4^, mean (range)	-	20.9 (5–39)	-	-
Parkinsonism, *n* (%)	47 (79.7%)	-	-	-
Abnormal Dat-Spect imaging, *n* (%)	55 (93.2%)	-	-	-

All ages and disease duration are expressed in years. ^1^ *p*, obtained by the Kruskal-Wallis test; ^2^ from the beginning of cognitive symptoms for DLB and AD, and motor symptoms in the case of PD; ^3^ MMSE, Mini-Mental State Examination; ^4^ UPDRs-III, Unified Parkinson’s disease rating scale.

**Table 3:** Relative expression of SNCA isoforms in four different brain regions and blood.

	SNCAtv1	SNCAtv2	SNCAtv3	SNCA112	SNCA126
FC ^1^	65.5% ^1^	22.3%	10.4%	0.5%	1.3%
^2^	52.2%	20.9%	20.9%	0.3%	0.7%
Ca ^3^	63.4%	25.3%	9.5%	0.6%	1.2%
Pt ^4^	63.1%	31.6%	3.4%	0.7%	1.2%
Blood	60.2%	20.4%	15.7%	1.2%	2.4%

^1^ FC, frontal cortex; ^2^ TC, temporal cortex; ^3^ Ca, caudate nucleus; ^4^ Pt, pons; blue: *SNCA* transcripts with different initial exons; orange: SNCA transcripts generated by in-frame splicing.

**Table 4:** Correlation SNCA transcript variant expression in LBD temporal cortices.

	SNCAtv1	SNCAtv2	SNCAtv3	SNCA126
**SNCAtv2**	**0.745** ***p* < 0.0001**			
**SNCAtv3**	−0.266 *p* = 0.141	−0.007 *p* = 0.934		
**SNCA126**	**0.715** ***p* < 0.0001**	**0.814** ***p* < 0.0001**	−0.202 *p* = 0.276	
**SNCA112**	**0.814** ***p* < 0.0001**	**0.834** ***p* < 0.0001**	−0.262 *p* = 0.162	**0.735** ***p* < 0.0001**

Pearson’s R-Scores and their corresponding *p*-values for each analysis. Significant results are marked in bold.

**Table 5:** Correlation SNCA transcript variant expression in blood of DLB and PD patients.

Correlation between	DLB	PD
SNCAtv1 and SNCAtv2	0.81 (*p* < 0.0001)	0.89 (*p* < 0.0001)
SNCAtv1 and SNCAtv3	0.44 (*p* = 0.0005)	0.77 (*p* < 0.0001)
SNCAtv2 and SNCAtv3	0.46 (*p* < 0.0001)	0.64 (*p* < 0.0001)
SNCAtv1 and SNCA126	0.75 (*p* < 0.0001)	0.57 (*p* < 0.0001)
SNCAtv2 and SNCA126	0.81 (*p* < 0.0001)	0.81 (*p* < 0.0001)
SNCAtv3 and SNCA126	0.81 (*p* < 0.0001)	0.81 (*p* < 0.0001)

Pearson’s R-Scores and their corresponding *p*-values for each analysis.

**Table 6:** cDNA primer sequences used for PCR amplification of SNCA transcript variants, neuron-specific enolase, synaptophysin, beta-actin and porphobilinogen deaminase.

Primer Name	Primer Sequence (5′–3′)	Primer Comb ^1^	Transcript ^2^
SNCA-1U *	ATC CAG GAA CAG CTG TCT TC	1U + 4L	SNCAtv3
SNCA-2aU *	TTC AAG CCT TCT GCC TTT CC	2aU + 4L	SNCAtv1
SNCA-2bU *	AGT CGG AGT TGT GGA GAA GCA	2bU + 4L	SNCAtv2
SNCA-4L	ACC ACT GCT CCT CCA ACA T		
SNCA-3/5U *	CTC TAT GTA GTG GCT GAG AA	3/5U + 6L	SNCA126
SNCA-4U	GTG CAT GGT GTG GCA ACA		
SNCA-5/7L *	ATA CCC TTC CTT GCC CAA C	3U + 5/7L	SNCA112
SNCA-6L	GAG CAC TTG TAC AGG ATG G		
NSE_6U	TGT ATC GCC ACA TTG CTC AG		
NSE_7L1	ACT GGG AGG ATC ATG AAC TC	6U + 7L1 ^3^	
NSE_7L2	ATG GCA TCC CGA AAG CTC TCA	6U + 7L2 ^3^	
SYPex2U	GCT TTG TGA AGG TGC TGC AAT	2U + 4L1 ^3^	
SYPex3U	TCT TCG CCA TCT TCG CCT TTG	3U + 4L1 ^3^	
SYPex4L1	TGC ATC AAA GTA CAC TTG GTG CA		
PBGD_U1	ACA CAC AGC CTA CTT TCC AAG		
PBGD_L1	TCA ATG TTG CCA CCA CAC TGT		
beta-actin U2	TCT ACA ATG AGC TGC GTG TG		
beta-actin L3	TAG ATG GGC ACA GTG TGG GT		

^1^ Primer comb, combination of primers used to amplify the different transcripts; ^2^ transcript, indicates which of the transcripts is amplified; ^3^ two fragments of each housekeeping gene, NSE and SYP amplified to assure reproducibility of the results; * these primers comprise transcript-specific sequences.

## Data Availability

Data is contained within the article. Raw data are available upon request to the Corresponding author.

## Abbreviations

DLB	dementia with Lewy bodies
PD	Parkinson’s disease
LBD	Lewy body disorders
LBP	Lewy body pathology
AD	Alzheimer disease
AS	alpha-synuclein
LB	Lewy body
PDD	PD dementia
CSF	cerebrospinal fluid
*SNCA*	alpha-synuclein gene
tv	transcript variant
UTR	untranslated region
CTRL	Control subjects
